# Motor Neurons with Axial Muscle Projections Specified by Wnt4/5 Signaling

**DOI:** 10.1016/j.neuron.2008.12.026

**Published:** 2009-03-12

**Authors:** Dritan Agalliu, Shinji Takada, Ilir Agalliu, Andrew P. McMahon, Thomas M. Jessell

**Affiliations:** 1 Howard Hughes Medical Institute, Kavli Institute for Brain Science, Departments of Neuroscience and Biochemistry and Molecular Biophysics, Columbia University Medical Center, New York, NY 10032, USA; 2 Department of Genetics and Development, Columbia University Medical Center, New York, NY 10032, USA; 3 Department of Molecular and Cellular Biology, Harvard Stem Cell Institute, Harvard University, Cambridge, MA 02138, USA; 4 Okazaki Institute for Integrative Biosciences, National Institutes of Natural Sciences, Okazaki 444-8787, Japan; 5 Department of Epidemiology and Population Health, Albert Einstein College of Medicine, Bronx, NY 10461, USA

**Keywords:** MOLNEURO, DEVBIO

## Abstract

Axial muscles are innervated by motor neurons of the median motor column (MMC). In contrast to the segmentally restricted motor columns that innervate limb, body wall, and neuronal targets, MMC neurons are generated along the entire length of the spinal cord. We show that the specification of MMC fate involves a dorsoventral signaling program mediated by three Wnt proteins (Wnt4, Wnt5a, and Wnt5b) expressed in and around the floor plate. These Wnts appear to establish a ventral^high^ to dorsal^low^ signaling gradient and promote MMC identity and connectivity by maintaining expression of the LIM homeodomain proteins Lhx3/4 in spinal motor neurons. Elevation of Wnt4/5 activity generates additional MMC neurons at the expense of other motor neuron columnar subtypes, whereas depletion of Wnt4/5 activity inhibits the production of MMC neurons. Thus, two dorsoventral signaling pathways, mediated by Shh and Wnt4/5, are required to establish an early binary divergence in motor neuron columnar identity.

## Introduction

Motor behaviors depend on the coordinate recruitment of different muscle groups, each activated by a specialized set of motor neurons. The activation of axial muscles controls many basic vertebrate motor programs. In aquatic vertebrates, axial muscles control the lateral undulations of the trunk and tail that underlie swimming, whereas in terrestrial vertebrates the axial musculature helps to stabilize the trunk during walking (O'Reilly et al., 2000; Deban and Carrier, 2002). The task of innervating axial muscles has been assigned to an evolutionarily conserved set of median motor column (MMC) neurons that are distinct, anatomically and functionally, from the motor neurons that innervate limb and body wall musculature (Fetcho, 1987, 1992).

Axial muscle innervation has its origins in the generation of subclasses of spinal motor neurons. Different classes of motor neurons are specified in modular fashion, through the actions of secreted signaling factors that assign diverse transcriptional codes to progenitor cells and postmitotic neurons (Shirasaki and Pfaff, 2002). The early specification of spinal motor neuron fate is initiated by a dorsoventral gradient of Sonic hedgehog (Shh) signaling activity (Jessell, 2000), which induces the sequential expression of a series of homeodomain (HD) transcription factors, notably the Nkx6.1/.2, Mnr2/Hb9, Lhx3/4, and Isl1/2 proteins, in ventral progenitors and postmitotic motor neurons (Dessaud et al., 2008). This dorsoventral signaling program operates along the entire length of the spinal cord, ensuring that motor neurons are produced at all segmental levels (Jessell, 2000).

**Figure 1:**
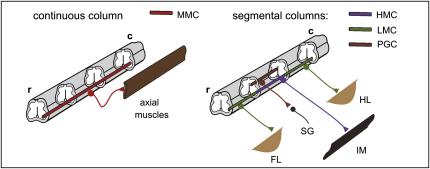
Motor Column Organization in the Spinal Cord Motor columns in the spinal cord. Median Motor Column (MMC, red) neurons are present at all segmental levels of the spinal cord and innervate axial musculature. Segmentally restricted motor columns (S-MC) are confined to discrete segmental levels. LMC neurons (green) project to limb musculature and are present at brachial and lumbar levels, whereas at thoracic levels hypaxial motor column neurons (HMC, purple) innervate body wall muscles, and preganglionic autonomic motor neurons (PGCs, brown) innervate sympathetic ganglia.

The diversification of this generic set of motor neurons depends on a second patterning system that operates along the rostrocaudal axis of the spinal cord and involves the graded signaling activities of fibroblast growth factors (FGFs) and retinoids (Liu et al., 2001; Sockanathan and Jessell, 1998; Dasen et al., 2003; Sockanathan et al., 2003). These extrinsic signals induce the expression of a network of Hox transcription factors whose collective activities specify motor neuron columnar classes at different segmental levels of the spinal cord (Figure 1) (Liu et al., 2001; Dasen et al., 2003, 2005, 2008). At brachial and lumbar levels, Hox activities direct the formation of lateral motor column (LMC) neurons, which project their axons to limb muscles (Landmesser, 1978; Lance-Jones and Landmesser, 1981; Dasen et al., 2003; Shah et al., 2004; Wu et al., 2008). At thoracic levels, preganglionic motor column (PGC) neurons innervating sympathetic neuronal targets are specified by Hox9 proteins (Markham and Vaughn, 1991; Prasad and Hollyday, 1991; Dasen et al., 2003), whereas hypaxial motor column (HMC) neurons innervating body wall muscles appear to be generated in a Hox-independent manner (Dasen et al., 2005, 2008; Rousso et al., 2008).

The general rule that motor neuron columnar classes are generated within segmentally restricted domains has one notable exception. Neurons of the median motor column (MMC) are found along the entire length of the spinal cord, a spatial profile that accommodates the need to innervate an iterated series of axial muscle groups (Figure 1) (Fetcho, 1987). As a consequence, nascent motor neurons at every segmental level of the spinal cord are faced with a basic decision choice about their fate: whether to generate MMC neurons or motor neurons destined to populate the other segmentally restricted motor columns.

At a transcriptional level, the assignment of MMC neuronal fate involves the postmitotic expression of two LIM homeodomain (HD) proteins, Lhx3 and Lhx4 (Shirasaki and Pfaff, 2002). Expression of the Lhx3/4 proteins renders motor neurons refractory to the segmental columnar patterning activities of Hox proteins (Tsuchida et al., 1994; Sharma et al., 2000; Dasen et al., 2003, 2005, 2008). Moreover, ectopic expression of Lhx3/4 proteins in spinal motor neurons is sufficient to reroute axons along a dorsal trajectory that brings them to axial muscles (Sharma et al., 2000). Thus, the Lhx3/4 proteins function as intrinsic determinants that impose the identity and connectivity of MMC neurons. Despite these advances, nothing is known about the early signaling events that ensure that a fraction of the motor neurons generated at each segmental level of the spinal cord progress to an MMC fate, rather than to segmentally restricted columnar subtypes.

The generation of MMC neurons along the entire rostrocaudal extent of the spinal cord prompted us to consider whether signals that operate along the dorsoventral axis specify MMC, as well as generic, motor neuron fate. The idea that dorsoventral signaling contributes to motor neuron diversification has received support from studies in the hindbrain showing that quantitative differences in the level of Shh signaling specifies the dorsoventral distinction between adjacent pMN and p3 progenitor domains that give rise to ventral (vMN) and dorsal (dMN) motor neuron classes (Ericson et al., 1997; Sharma et al., 1998; Lieberam et al., 2005). Yet in the spinal cord, all motor neurons derive from the pMN domain (Ericson et al., 1997; Jessell, 2000). Thus, it remains unclear whether dorsoventral signaling has any role in spinal motor neuron diversification. And if it does, is Shh or another as yet unidentified signaling factor responsible for this patterning activity?

To address these issues, we set out to examine whether inductive signals that operate along the dorsoventral axis of the spinal cord regulate the developmental decision to generate MMC neurons. Using a combination of molecular and genetic methods in chick and mouse embryos, we show that the generation of MMC neurons depends on the dorsoventral position at which motor neurons are generated—MMC neurons can be induced ventral, but not dorsal, to the normal position of motor neuron generation. The position-dependent nature of MMC generation can be traced to the activities of a triumvirate of Wnt genes expressed in and around the floor plate. Wnt4, Wnt5a, and Wnt5b act redundantly to establish a ventral^high^ to dorsal^low^ signaling gradient that specifies MMC identity and connectivity by promoting persistent expression of Lhx3/4 in postmitotic motor neurons. Elevation of Wnt4/5 activity results in the generation of additional MMC neurons, at the expense of HMC and LMC neurons, whereas depletion of Wnt4/5 activity inhibits the production of MMC neurons and generates additional HMC neurons. Together, our findings show that motor neuron generation in the spinal cord depends on two dorsoventral signaling systems—mediated by Shh and Wnt4/5 proteins—and that the concerted activity of these two systems establishes an early divergence in motor neuron columnar identity.

## Results

### Dorsoventral Position of Motor Neuron Generation Influences MMC Fate

We first examined whether the specification of MMC neuronal identity is influenced by the dorsoventral position of motor neuron generation within the spinal cord. To explore this issue, we used loss- and gain-of-function approaches in mouse and chick to elicit motor neuron differentiation at ectopic ventral or dorsal positions and assessed the columnar identity of the supernumerary, misplaced, motor neurons. MMC neurons were defined by coexpression of the HD proteins Lhx3/4, Isl1/2, and Hb9; HMC neurons by coexpression of Isl1/2 and Hb9 in the absence of Lhx3/4; and PGC neurons by expression of Isl1 in the absence of other HD proteins, as well as by their dorsal settling position (Tsuchida et al., 1994; Dasen et al., 2003). At limb levels, medial LMC neurons were defined by Isl1 expression in the absence of Lhx3/4 and Hb9, and lateral LMC neurons by coexpression of Isl2 and Hb9 (Tsuchida et al., 1994; Arber et al., 1999; Thaler et al., 1999).

To elicit motor neuron generation at an ectopic ventral position, we examined mice mutant for the homeobox gene *Nkx2.2*. In *Nkx2.2* mutants, progenitor cells in the p3 domain, which lies ventral to the normal domain of motor neuron differentiation, switch their fate from p3 to pMN identity, with the consequence that motor neurons rather than V3 neurons are generated (Briscoe et al., 1999). The columnar subtype identity of these ectopic motor neurons has not been resolved, however. We therefore quantified the number and columnar subtype of motor neurons in wild-type and *Nkx2.2* mutant mice at e13.5, after columnar identities have been consolidated (Briscoe et al., 1999).

**Figure 2:**
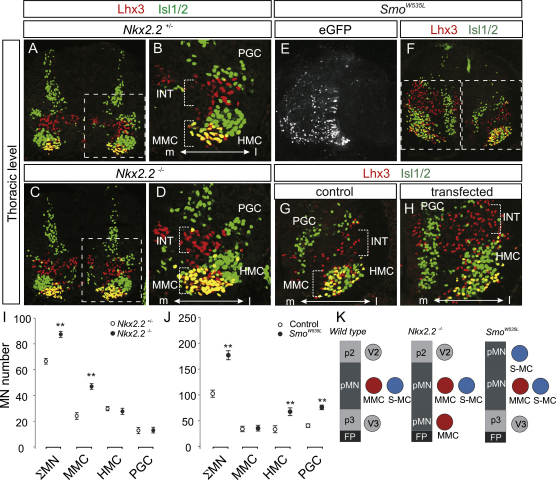
MMC Fate as a Function of the Dorsoventral Position of Motor Neuron Generation (A–D) Lhx3 (red) and Isl1/2 (green) expression in thoracic spinal cord from *Nkx2.2*^+/−^ (A and B) and *Nkx2.2*^−/−^ (C and D) mice at e13.5. Isl1/2^on^ neurons in the ventral spinal cord are motor neurons (MNs); Lhx3^on^, Isl1/2^on^ cells are MMC neurons; and Lhx3^on^, Isl1/2^off^ neurons are V2a interneurons. Note the increase in MMC neuron number (yellow) in *Nkx2.2* mutants. PGC neurons are located dorsolaterally. (E–H) In ovo electroporation of a constitutively active *Smoothened* receptor (*Smo^W535L^*) in chick spinal cord is revealed by marker eGFP expression (E). (F–H) Lhx3 (red) and Isl1/2 (green) expression in transfected thoracic chick spinal cord (HH stage 29). PGC neurons are located dorsomedially in the chick. (I) Plots of motor neuron numbers per ventral 15 μm quadrant in *Nkx2.2*^+/−^ (open circles) and *Nkx2.2*^−/−^ embryos (closed circles); mean ± SEM (n = 5 embryos per genotype, Student's t test, ^∗∗^p < 0.01). (J) Plot of motor neuron numbers for *Smo^W535L^*-transfected embryos (mean ± SEM; n = 8 embryos, Student's t test, ^∗∗^p < 0.01, open circles represent controls and closed circles *Smo^W535L^*-transfected sides). (K) Summary of ectopic generation of motor neurons along the dorsoventral axis of the spinal cord in *Nkx2.2^−/−^* mutant mouse and *Smo^W535L^* chick embryos.

At thoracic spinal levels of e13.5 wild-type mice, the total cohort of motor neurons (mean: 66 motor neurons/ventral quadrant/15 μm section) comprised ∼35% MMC neurons, ∼40% HMC neurons, and ∼25% PGC neurons (Figures 2A, 2B, and 2I). At brachial and lumbar levels, the total motor neuron cohort (mean brachial: 125 motor neurons/ventral quadrant/15 μm section; mean lumbar: 138 motor neurons/ventral quadrant/15 μm section) comprised ∼20% MMC and ∼80% LMC neurons (Figure S1A, S1B, S1E, S1F, S1I, and S1J available online). In *Nkx2.2* mutants, there was an ∼30% increase in total motor neuron number at thoracic levels and an ∼20% increase at brachial and lumbar levels (Figures 2I, S1I, and S1J). We detected an ∼2-fold increase in the number of MMC neurons at brachial, thoracic, and lumbar levels of *Nkx2.2* mutants, whereas the number of HMC, PGC, and LMC neurons was unchanged (Figures 2C, 2D, 2I, S1C, S1D, and S1G–S1J). Quantitatively, the increase in total motor neuron number in *Nkx2.2* mutants could be accounted for, in its entirety, by the increase in MMC number (Figures 2I, S1I, and S1J). These findings indicate that all of the additional motor neurons generated from an ectopic ventral position in *Nkx2.2* mutants acquire an MMC identity.

To induce the differentiation of motor neurons at ectopic dorsal positions, we used in ovo electroporation in chick spinal cord to express an isoform of the Shh receptor subunit, Smoothened (Smo^W535L^), which activates the Shh transduction pathway constitutively and in a cell-autonomous manner (Hynes et al., 2000). Stage 12–14 chick thoracic neural tube was electroporated unilaterally with a *Smo^W535L^::IRES::nGFP* construct and the identity of GFP-labeled progenitors and postmitotic motor neurons analyzed between stages 21 and 30. Expression of *Smo^W535L^* resulted in a dorsal expansion of the domain occupied by Olig2^+^ pMN domain progenitors and an ∼2-fold increase in total number of Olig2^+^ progenitor cells at stages 21 to 24 (Figures S2A, S2B, and S2E). Expression of *Smo^W535L^* also elicited a 1.7-fold increase in the number of Isl1/2^+^ motor neurons at stages 29 to 30 (p < 0.01 versus controls) (Figures S2H and S2J). In addition, *Smo^W535L^* expression induced the ectopic dorsal differentiation of V2a and V3 neurons, two interneuron classes that derive from the p2 and p3 progenitor domains that flank, dorsally and ventrally, the position of motor neuron generation (Figures S2G, S2I, and S2J) (Briscoe et al., 2000). The induction of V2a neurons, motor neurons, and V3 neurons in response to *Smo^W535L^* expression presumably reflects variation in the level of activation of the Shh transduction pathway in individual progenitor cells.

We analyzed the columnar identity of motor neurons generated at ectopic dorsal positions in the thoracic spinal cord. The number of MMC neurons was unchanged after *Smo^W535L^* expression (p > 0.05, versus control side). In contrast, the number of HMC neurons increased ∼2.5 fold, and the number of PGC neurons increased ∼2 fold (Figures 2E–2H and 2J) (p < 0.01 versus controls). Thus, few, if any, of the ectopic dorsal motor neurons induced by *Smo^W535L^* acquire an MMC identity, despite activation of the Shh transduction pathway at levels that span the range sufficient for motor neuron induction. Together, these findings show that cell position along the dorsoventral axis of the spinal cord has a marked influence on the probability of generation of MMC neurons (Figure 2K).

### Patterned Expression of *Wnt* Genes in the Ventral Spinal Cord

We next considered the possible source and identity of extrinsic signals that specify MMC neuronal subtype. Since MMC differentiation is highly sensitive to dorsoventral position, we considered whether the cells of the floor plate or adjacent ventral neural tube might serve as a source of relevant inductive signals. Our data suggest that Shh signaling alone is insufficient to specify MMC fate, prompting us to examine other candidate signals. We focused on Wnt proteins because of their known expression in the ventral spinal cord (Parr et al., 1993; Hollyday et al., 1995) and their ability to induce expression of a key MMC transcriptional determinant, Lhx3, in neuroendocrine cells (Satou et al., 2001). We analyzed the expression of Wnts as well as soluble frizzled-related proteins (Sfrps)—a class of secreted Wnt-binding proteins that inhibit Wnt signaling—and the Frizzled (Fz) class of Wnt receptors (Kawano and Kypta, 2003; Gordon and Nusse, 2006).

We examined the expression of 17 Wnt genes in the ventral spinal cord of e9.5 to e11.5 mouse embryos, the peak period of motor neuron generation. Wnt ligands have been assigned to three main classes on the basis of their signal transduction pathways. Wnt1, -3, and -8 are strong activators, and Wnt7 proteins weak activators of the β-catenin transduction pathway, whereas Wnt4/5 proteins typically fail to activate β-catenin transduction, instead engage PKC, CamKII, or intracellular Ca^2+^ signaling pathways (Veeman et al., 2003; Gordon and Nusse, 2006).

**Figure 3:**
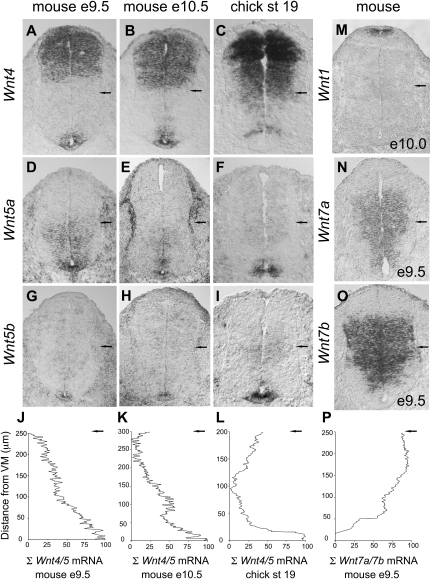
Expression of Wnt Genes in the Ventral Spinal Cord (A–I) Expression of *Wnt4* (A–C), *Wnt5a* (D–F), and *Wnt5b* (G–I) in the ventral spinal cord of e9.5 and e10.5 mouse embryos and HH stage 19–20 chick embryos. *Wnt4* is expressed in the floor plate, the p3 domain, and the dorsal spinal cord in the mouse (A and B) but not in the chick floor plate (C). *Wnt5a* is expressed broadly in the ventral spinal cord of e9.5 and e10.5 mouse embryos (D and E) but is largely restricted to the floor plate and p3 domain in chick embryos (F). *Wnt5b* is restricted to the floor plate in mouse and chick spinal cord (G–I). (J–L) Plots of W*nt4/5a/5b* transcript expression level in the ventral spinal cord of e9.5 mouse (J), e10.5 mouse (K), and chick (L) embryos. (M–O) W*nt1* (M) expression in e10.0 mouse spinal cord and *Wnt7a* (J) and *Wnt7b* (K) expression in e9.5 mouse spinal cord. *Wnt1* is expressed in the roof plate. *Wnt7a* and *Wnt7b* are present in the ventral spinal cord but are excluded from the floor plate and p3 domain. (P) Plots of *Wnt7a* and *Wnt7b* transcripts in the ventral spinal cord of e9.5 mouse embryos. In all plots, the x axis represents transcript levels and the y axis distance (μm) from the ventral midline to intermediate spinal cord (black arrow).

*Wnt1*, -*3*, and -*3a* were expressed exclusively in the dorsal spinal cord (Figure 3M; data not shown; see Parr et al., 1993). Members of the Wnt8 family (*Wnt8a*, -*8b*, and -*8c*) were not expressed in chick or mouse spinal cord (data not shown; see Parr et al., 1993). *Wnt7a* and -*7b* were expressed in a dorsal^high^ to ventral^low^ gradient within the ventral spinal cord between e9.5 and e10.5 (Figures 3N–3P). The level of expression of *Wnt7b* appeared greater than that of *Wnt7a*, but the ventral boundary of *Wnt7a* extended more ventrally than that of *Wnt7b*, approaching the floor plate (Figures 3N and 3O; Lei et al., 2006).

In contrast, *Wnt4*, *-5a*, and -*5b* were expressed in a ventral^high^ to dorsal^low^ gradient within the ventral spinal cord of mouse embryos. *Wnt4* was expressed at high levels in the floor plate and p3 domain (Figures 3A and 3B) (Parr et al., 1993), and its expression level decreased in more dorsal regions. *Wnt4* was also expressed at high levels in the dorsal spinal cord (Figures 3A and 3B) (Parr et al., 1993). At e9.5, *Wnt5a* was expressed at high levels by progenitor cells throughout the ventral spinal cord, but by e10.5 became largely restricted to the floor plate and pMN domain (Figures 3D and 3E). *Wnt5b* was expressed by the floor plate between e9.5 and e10.5 (Figures 3G and 3H). Quantitatively, analysis of the cumulative level of *Wnt4*, *-5a*, and *-5b* transcripts in the ventral spinal cord of mouse embryos revealed a ventral^high^ to dorsal^low^ expression gradient at e9.5 and e10.5 (Figures 3J and 3K).

We also analyzed the expression of Wnt ligands in chick spinal cord between stages 18 to 24, the peak period of motor neuron generation. The patterns of expression of *Wnt1*, *-3*, *-3a*, *-7a*, and *-7b* were similar to those in mouse (data not shown; Hollyday et al., 1995). *Wnt4* was not expressed by the floor plate but was detected at high levels in the p3 progenitor domain and in the dorsal spinal cord (Figure 3C) (Hollyday et al., 1995). *Wnt5a* and -*5b* were expressed at high levels in the floor plate and at lower levels within the p3 domain (Figures 3F and 3I). The cumulative distribution of chick *Wnt4*, -*5a*, and -*5b* transcripts within the ventral spinal cord revealed a ventral^high^ to dorsal^low^ expression gradient comparable to that observed in mouse embryos (Figure 3L).

We next examined the expression of frizzled class Wnt receptors and secreted frizzled related proteins (Sfrps). Four *Fz* genes were expressed in the ventral spinal cord over the period of motor neuron generation. *Fz2* and *Fz7* were expressed by neural progenitors, in both mouse and chick (Figures S3D and S3E), with near-uniform levels of expression along the dorsoventral axis of the spinal cord (Figure S3F). *Fz9* was detected in mouse, but not chick, progenitor cells (data not shown), and *Fz3* was expressed broadly in both progenitor cells and postmitotic neurons (data not shown; Lyuksyutova et al., 2003). Other *Fz* genes were not detected in ventral progenitors or motor neurons at these developmental stages (data not shown). A high level of *Sfrp2* was detected in the ventral spinal cord at e9.5 to e10.5, with a ventral boundary at the interface of the p3 and pMN domains (Figure S3B; data not shown; Lei et al., 2006). The pattern of *Sfrp1* expression was similar to that of *Sfrp2*, although low levels of expression were also detected within the p3 domain at e9.5 (Figure S3A; data not shown). The cumulative profile of *Sfrp1/2* expression along the dorsoventral axis of the ventral spinal cord was inverted when compared to that of *Wnt4*, *Wnt5a*, and *Wnt5b* transcripts, exhibiting a dorsal^high^ to ventral^low^ expression gradient (Figure S3C).

### Wnt4 and Wnt5 Promote MMC Columnar Identity

The inverse dorsoventral gradients of *Wnt7a*/*7b* and *Wnt4*/*5a*/*5b* expression led us to test whether the specification of MMC neurons results from the evasion of dorsally derived Wnt7 ligands or from exposure to ventrally derived Wnt4/5 ligands. To resolve this issue, we explored whether any of these Wnts have MMC-inducing activity in chick spinal cord in vivo. We used in ovo electroporation to coexpress *Wnt* cDNAs together with a marker *eGFP* construct in the ventral spinal cord of stage 12–14 chick embryos and assessed the columnar identity of motor neurons by their transcriptional profile and axonal projection pattern at stages 26–29. We focused on thoracic and lumbar levels of the spinal cord for this analysis because, at these more caudal levels, transgene expression can be achieved at an earlier stage of neuronal differentiation. The activities of *Wnt4*, *Wnt5a*, *Wnt5b*, and *Wnt7b* were compared with that of *Wnt1*, a strong activator of β-catenin transduction.

**Figure 4:**
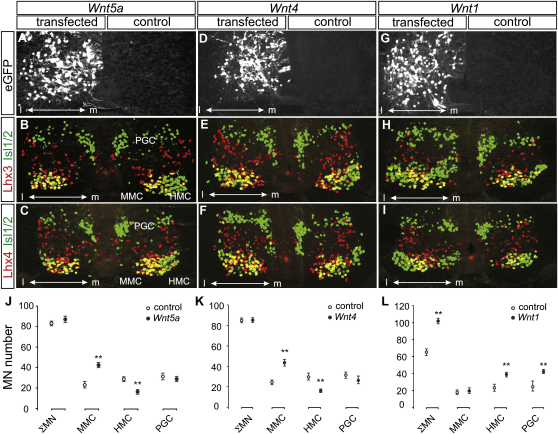
Misexpression of Wnt4/5 Genes Specifies MMC Columnar Identity Electroporation of stage 12–14 chick thoracic spinal cord with *Wnt5a* (A–C and J), *Wnt4* (D–F and K) or *Wnt1* (G–I and L). Tracer eGFP expression (A, D, and G) labels the transfected side of the spinal cord. Different combinations of Lhx3 (red) and Isl1/2 (green) expression mark MMC, HMC, and PGC neurons (B, E, and H). Thoracic misexpression of W*nt5a* does not change total motor neuron number, but leads to an increase in the number of MMC neurons and to a decrease in HMC neurons (A–C and J). *Wnt4* and *Wnt5b* (not shown) transfection produces effects similar to *Wnt5a* (D–F and K). Ectopic MMC neurons also express Lhx4 (C and F). Transfection of *Wnt1* increases the total number of motor neurons, but not the number of MMC neurons (H). Note the aberrant migration of PGC neurons in thoracic segments of *Wnt1*-transfected embryos (H and I). *Wnt4*- and *Wnt5a*-induced ectopic MMC neurons also express Lhx4 (C and F). (J–L) Plots of motor neuron columnar subtype per ventral quadrant 15 μm section in *Wnt5a* (J), *Wnt4* (K), and *Wnt1* (L) transfected embryos, mean ± SEM (Student's t test, ^∗∗^p < 0.01; ^∗^p < 0.05; n = 20 embryos for *Wnt5a*, n = 16 embryos for *Wnt4*, and n = 10 embryos for *Wnt1* transfection).

Expression of *Wnt4*, *Wnt5a*, or *Wnt5b* did not change the number of Olig2^+^ motor neuron progenitors (Figures S4A–S4D and S4G; data not shown), nor was there a change in motor neuron number at brachial, thoracic, or lumbar levels of the spinal cord (Figures 4J, 4K, S5E, S6J, and S6K; data not shown). Nevertheless, *Wnt4*, -*5a*, or -*5b* expression elicited a 1.7- to 2.0- fold increase in the number of MMC neurons at thoracic levels, assessed by coexpression of Lhx3/4 and Isl1/2 (p < 0.01; Student's t test; n = 16–20 embryos for each Wnt assayed) (Figures 4A–4F, 4J, 4K, S5A, S5B, and S5E). The increase in MMC neurons was accompanied by a 2-fold decrease in the number of HMC neurons (p < 0.01; Student's t test; n = 16–20 embryos), whereas the number of PGC neurons was not changed (Figures 4A–4F, 4J, 4K, S5A, S5B, and S5E). Expression of *Wnt4* and Wnt*5a*, the two Wnts analyzed at lumbar levels, increased the number of MMC neurons ∼2.7 fold, at the expense of LMC neurons which exhibited a small decrease in number (p < 0.05; Student's t test; n = 16–20 embryos) (Figures S6A–S6F, S6J, and S6K). We also examined whether there is a temporal constraint on Wnt4/5 signaling activity. We found that thoracic or lumbar electroporation of *Wnt4*, -*5a*, or -*5b* at stage 18, rather than stage 12–14, failed to elicit a change in motor neuron columnar identities (data not shown). Thus, early, but not late, expression of each of three noncanonical Wnts in the ventral spinal cord enhances the generation of MMC neurons, at the expense of other motor neuron columnar subtypes.

**Figure 5:**
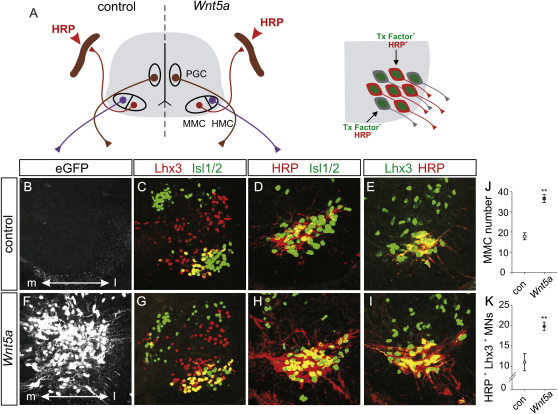
MMC Neurons Induced by Wnt5a Project to Axial Muscles (A) HRP labeling of MMC neurons after tracer injection into axial muscles in control or *Wnt5a*-transfected chick spinal cords. (B–I) eGFP expression marks the electroporated side of the spinal cord (B and F), Lhx3 and Isl1/2 expression (C and G), HRP labeling of Isl1/2^on^ neurons (D and H) HRP labeling of Lhx3^on^ neurons (E and I) in control and *Wnt5a*-electroporated thoracic spinal cord. There is an increase in the number of MMC neurons (G) and the number of HRP-labeled Lhx3^on^ Isl1/2^on^ motor neurons (H and I) in *Wnt5a*-transfected embryos. (J and K) Plots of MMC neurons (J) and HRP^on^, Lhx3^on^ motor neurons (K) in controls (open circle) or *Wnt5a-*transfected embryos (closed circle). Circles with bars represent mean ± SEM (Student's t test, ^∗∗^p < 0.01, n = 8 embryos).

We also determined if the axons of Lhx3/4^+^ motor neurons induced by *Wnt4/5* signaling pursue a trajectory that is consistent with their apparent MMC character. To assess this, we monitored the transcriptional status of retrogradely labeled motor neurons in the thoracic spinal cord of stage 29–30 chick embryos after injection of horseradish peroxidase (HRP) into axial muscles (Figure 5A). On the control side of the spinal cord, ∼60% of all Lhx3^on^ MMC neurons accumulated HRP, and more importantly, all HRP-labeled neurons expressed Lhx3 (Figures 5B–5E, 5J, and 5K). On the side of the spinal cord transfected with *Wnt5a*, we detected an ∼2.1-fold increase in the total number of MMC neurons (Figures 5G and 5J). We found that ∼55% of all Lhx3^on^ motor neurons accumulated HRP, a proportion similar to that found in controls (p = 0.58) (Figures 5F–5I and 5K). Furthermore, all HRP-labeled motor neurons expressed Lhx3 (Figures 5F–5I). Together, these findings indicate that the extra Lhx3^on^ neurons generated in response to enhanced *Wnt4/5* expression send their axons along a dorsal branch that takes them to axial muscles—the trajectory and target of MMC neurons.

Wnt1 signaling has been reported to regulate the pattern of homeodomain transcription factors in ventral progenitor cells (Lei et al., 2006; Alvarez-Medina et al., 2008; Yu et al., 2008), prompting us to examine whether Wnt1 activity influences the specification of MMC neurons. We found that ectopic expression of *Wnt1* in the ventral spinal cord results in an ∼2-fold increase in the number of Olig2^+^ progenitor cells (Figures S4E–S4G) and an ∼1.5 fold increase in the number of postmitotic motor neurons (both p < 0.01 versus controls, Student's t test; n = 10 embryos) (Figures 4G–4I, 4L, S6G–S6I, and S6L). Similar Wnt1 inductive activities were observed at thoracic and lumbar levels of the spinal cord. At thoracic levels, *Wnt1* expression did not change the number of MMC neurons (p > 0.05; Student's t test; n = 10 embryos), but led to an ∼1.7-fold increase in the number of HMC and PGC neurons (p < 0.01, Student's t test; n = 10 embryos) (Figures 4G–4I and 4L). Similarly, at lumbar levels, *Wnt1* expression did not change the number of MMC neurons but led to an ∼1.5 fold increase in the number of LMC neurons (Figures S6G–S6I and S6L). Neurons in the medial and lateral divisions of the LMC exhibited a similar increase in neuronal number (p < 0.01; Student's t test; n = 10 embryos; data not shown). The selective increase in PGC, HMC, and LMC neurons upon *Wnt1* expression may have its basis in the enhanced proliferation of ventral progenitor cells (Megason and McMahon, 2002), such that most of the additional motor neurons are generated at more dorsal positions, beyond the range of Wnt4/5 signaling (Figures S4E and S4F). Expression of *Wnt7b*, in contrast, did not change the number of Olig2^+^ motor neuron progenitors or total motor neuron number (Figure S5F; data not shown). Furthermore, the fraction of motor neurons allocated to individual motor columns was not altered by thoracic or lumbar *Wnt7b* expression (Figures S5C, S5D, and S5F; data not shown). Thus, *Wnt4/5* but not *Wnt1* or *Wnt7b* activities enhance the generation of MMC neurons at the expense of other motor neuron columnar subtypes.

### Persistence of MMC Identity after Disruption of the Wnt Planar Polarity Pathway

We attempted to clarify the neural signaling pathway that links Wnt4/5 activity to the maintenance of Lhx3 expression. The observation that Wnt1 fails to mimic the MMC-inducing activity of Wnt4/5 argues against the involvement of the β-catenin pathway (Gordon and Nusse, 2006). In certain cellular contexts, noncanonical Wnt ligands, including Wnt4/5, interact with Fz3, Fz5, and Fz7 receptors (He et al., 1997; Lyuksyutova et al., 2003; Veeman et al., 2003). We therefore examined whether overexpression of Ig-modified versions of the CRD ectodomains of Fz5 and Fz7 (Hsieh et al., 1999) in embryonic chick spinal cord is able to block MMC specification. We found, however, that both these reagents severely reduced the total number of motor neurons (data not shown), precluding a meaningful analysis of MMC specification. The reduction in motor neuron generation is likely to reflect the blockade of canonical Wnts that promote cell proliferation in the ventral neural tube (Megason and McMahon, 2002).

Many noncanonical Wnt ligands activate the vertebrate planar cell polarity pathway, a transduction system that depends on the function of Vangl2/Ltap, a vertebrate homolog of the *Drosophila* Van Gogh/Strabismus protein (Kibar et al., 2001). We therefore considered whether Wnt4/5 induction of MMC identity involves this signaling pathway, analyzing motor neuron differentiation in *loop tail* mutant mice (which carry a null mutation in the *Vangl2/Ltap* gene) (Kibar et al., 2001). At e13.5, the total number of motor neurons and the proportional allocation of MMC neurons were similar in the thoracic spinal cord of wild-type and *loop tail* embryos (Figures S7A–S7G) (p > 0.05; Student's t test; n = 3 embryos), arguing against the involvement of the Wnt planar cell polarity pathway in MMC specification.

### Switch from MMC to Segmental Columnar Subtypes in *Wnt4/5* Mutant Mice

To address the requirement for Wnt4/5 signaling in the specification of MMC identity, we used mouse genetics to examine the impact of reducing Wnt activity on the assignment of motor neuron columnar identities. We assessed motor neuron columnar identity in 14 of 27 possible *Wnt4*, -*5a*, and -*5b* allelic combinations, eliminating from one to five *Wnt* alleles (Stark et al., 1994; Yamaguchi et al., 1999). We found that *Wnt4^−/−^; Wnt5a^−/−^; Wnt5b^−/−^* triple-mutant embryos died before the onset of motor neuron differentiation (data not shown). *Wnt5a* mutants exhibit severe defects in limb development (Yamaguchi et al., 1999) that are likely to perturb motor neuron differentiation at limb levels of the spinal cord through other cellular mechanisms (Oppenheim et al., 1986; Lanser and Fallon, 1987). For this reason, we focused our analysis primarily on thoracic levels, assessing the impact of progressive removal of *Wnt* alleles on motor neuron differentiation.

**Figure 6:**
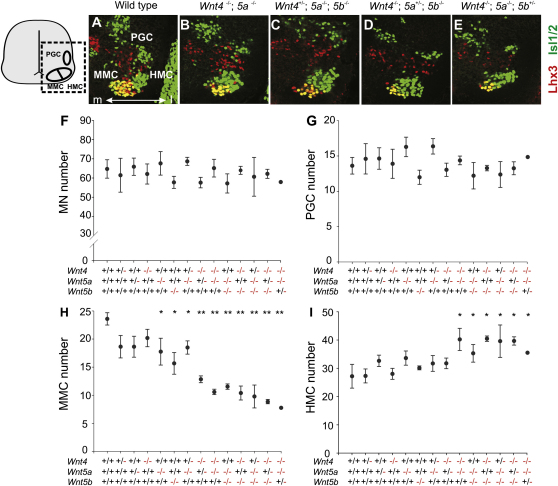
Thoracic Motor Neuron Columnar Subtypes in *Wnt4/5* Mutant Mice (A–E) Lhx3 and Isl1/2 expression in e13.5 thoracic spinal cord from wild-type and allelic combinations of *Wnt4*, *Wnt5a*, and *Wnt5b* mutant mice. Isl1/2^on^ ventral neurons are motor neurons, and Lhx3^on^ Isl1/2^on^ neurons (yellow) are MMC neurons. (F–I) Plots representing the total number of motor neurons (F), PGC neurons (G), MMC neurons (H), and HMC neurons (I) at thoracic levels in various mutant *Wnt4*, *Wnt5a*, and *Wnt5b* allelic combinations; mean ± SEM (ANOVA test, ^∗∗^p < 0.01, ^∗^p < 0.05, n = 3–6 embryos for different genotypes, except *Wnt4^−/−^; Wnt5a^−/−^; Wnt5b^+/−^* [n = 1]).

Mice heterozygous for *Wnt4*, *Wnt5a*, or *Wnt5b* did not show a significant difference in thoracic motor neuron number, nor in the fractional representation of motor columnar subtypes when compared to wild-type embryos (Figures 6F–6I). Similarly, we found that *Wnt4*, *Wnt5a*, or *Wnt5b* single-mutant embryos exhibited no significant difference in thoracic motor neuron number, nor in the representation of motor columnar subtypes when compared with wild-type or heterozygous embryos (Figures 6F–6I).

Analysis of mice carrying three mutated *Wnt* alleles (*Wnt4^−/−^; Wnt5a^+/−^* and *Wnt4^+/−^; Wnt5a^−/−^* genotypes) revealed that the total number of motor neurons was unchanged (Figures 6F). In *Wnt4^−/−^; Wnt5a^+/−^* mice, we detected a 46% decrease in the number of MMC neurons (24 ± 2.0 neurons in wild-types versus 12.7 ± 2.5 in *Wnt4^−/−^; Wnt5a^+/−^* embryos; ANOVA test; p < 0.01; n = 6 embryos) (Figure 6H). The reduction in MMC neuronal number was accompanied by an increase in the number of HMC neurons (26 ± 4.0 neurons in wild-types versus 32 ± 3.5 in *Wnt4^−/−^; Wnt5a^+/−^* embryos; ANOVA test; p = 0.02; n = 6 embryos), whereas the number of PGC neurons was unchanged (Figures 6G and 6I). Analysis of *Wnt4^+/−^; Wnt5a^−/−^* mutants revealed a smaller (22%) decrease in the number of MMC neurons (24 ± 2.0 neurons in wild-types versus 17.8 ± 2.4 in *Wnt4^+/−^; Wnt5a^−/−^* mutants; ANOVA test; p = 0.022; n = 5 embryos), and there was no significant change in the representation of other columnar subtypes (Figures 6G–6I).

In *Wnt4^−/−^; Wnt5a^−/−^*, *Wnt5a^−/−^; Wnt5b^−/−^*, and *Wnt4^−/−^; Wnt5b^−/−^* double mutant embryos, there was no change in thoracic motor neuron number, but a more marked (50%–60%) decrease in the number of MMC neurons (58% decrease in *Wnt4^−/−^; Wnt5a^−/−^*, 58% decrease in *Wnt4^−/−^; Wnt5b^−/−^*, and 50% decrease in *Wnt5a^−/−^; Wnt5b^−/−^* genotypes, compared to wild-type; ANOVA test; p < 0.01; n = 3–5 embryos per genotype) (Figures 6B and 6H). Conversely, there was a significant increase in the number HMC neurons (40 ± 4 neurons in *Wnt4^−/−^; Wnt5a^−/−^*, 35 ± 4 neurons in *Wnt5a^−/−^; Wnt5b^−/−^*, and 40 ± 4 neurons in *Wnt4^−/−^; Wnt5b^−/−^* versus 26 ± 4 neurons in wild-type littermates; ANOVA test; p < 0.05; n = 3–5 embryos per genotype) (Figures 6B and 6I). These findings provide evidence that *Wnt4*, *Wnt5a*, and *Wnt5b* each contribute to the specification of MMC identity.

Each of the three combinations of five mutated *Wnt* alleles exhibited a marked decrease in MMC neuronal number, compared to wild-type controls (59% in *Wnt4^+/−^; Wnt5a^−/−^; Wnt5b^−/−^*, 63% in *Wnt4^−/−^; Wnt5a^+/−^; Wnt5b^−/−^*, and 67% in *Wnt4^−/−^; Wnt5a^−/−^; Wnt5b^+/−^*; ANOVA test; p < 0.01) (Figures 6C–6E and 6H). In addition, there was a compensatory increase in HMC neuronal number (62% in *Wnt4^+/−^; Wnt5a^−/−^; Wnt5b^−/−^*, 50% in *Wnt4^−/−^; Wnt5a^+/−^; Wnt5b^−/−^*, and 39% in *Wnt4^−/−^; Wnt5a^−/−^; Wnt5b^+/−^*; ANOVA test; p < 0.01) (Figures 6C–6E and 6I). In contrast, the number of PGC neurons was unchanged (Figure 6G). These findings provide genetic evidence that, at thoracic levels, prospective MMC neurons switch to an alternate, HMC, fate in the absence of Wnt4/5a/5b signaling.

We also analyzed the impact of eliminating Wnt4/5 signaling on motor columnar identity at lumbar levels of the spinal cord. As discussed, this analysis was complicated by the fact that loss of Wnt4/5 signaling impairs limb development, with potential secondary consequences for motor neuron differentiation and survival. Indeed, embryos carrying four or five mutated *Wnt4/5* alleles exhibited a small (∼20%) reduction in total motor neuron number at hindlimb levels, compared to wild-type controls (ANOVA test, p < 0.01 versus controls) (Figure S8A). Nevertheless, we detected a greater (∼50%) decrease in the fraction of MMC neurons at lumbar levels of *Wnt4^−/−^; Wnt5b^−/−^* and *Wnt4^−/−^; Wnt5a^+/−^; Wnt5b^−/−^* mutants (ANOVA test; p < 0.01 versus controls; n = 3 embryos) (Figure S8B). These findings support the view that Wnt4, Wnt5a, and Wnt5b signaling promotes the generation of MMC neurons at limb as well as thoracic levels of the spinal cord.

## Discussion

Vertebrates have discovered many uses for axial muscles, each of which depends on their activation by spinal motor neurons located in the MMC. But the fundamental issue of how motor neurons acquire an MMC identity that ensures axial muscle activation has not been resolved. We have found that Wnt4/5 expression by cells in and adjacent to the ventral midline of the spinal cord promotes the progression of nascent motor neurons to an MMC fate. Our findings reveal the existence of two parallel ventrodorsal signaling gradients, mediated by Hh and Wnt proteins, and show that these two signaling pathways have convergent functions in specifying the identity and connectivity of spinal motor neurons. Wnt4 signaling has also been shown to direct the rostral trajectory of the axons of spinal commissural neurons, after their passage through the floor plate (Lyuksyutova et al., 2003). Noncanonical Wnts therefore join BMPs (Augsburger et al., 1999; Butler and Dodd, 2003) and Shh (Charron et al., 2003) as versatile signaling factors that specify both neuronal fate and axonal trajectory in the developing spinal cord.

### Wnt4/5 Signaling Promotes MMC Identity

**Figure 7:**
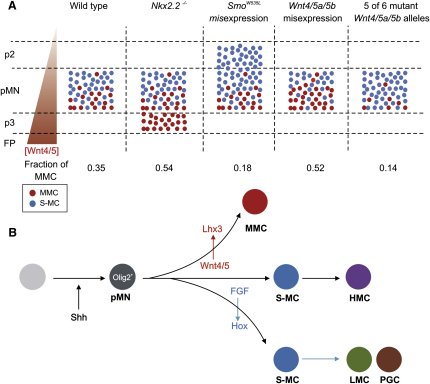
Wnt4/5 Signaling and the Specification of MMC Identity (A) Fractional representation of MMC neurons in various experimental conditions, with quantification derived from our thoracic-level data. *Wnt4*, *Wnt5a*, and *Wnt5b* are distributed in a ventral^high^ to dorsal^low^ gradient in the ventral spinal cord (red triangle). Model shows a scenario in which cells located in more ventral regions of the pMN domain have a higher probability of acquiring MMC identity (red cells), whereas cells located at more dorsal regions of the pMN domain are more likely to acquire segmental column fates (S-MC, blue cells). This model is supported by the finding that in *Nkx2.2*^−/−^ mice all motor neurons generated from the p3 domain acquire MMC identity, whereas in *Smo^W535L^* chick embryos, neurons generated at positions dorsal to the pMN domain acquire segmental columnar fates. Misexpression of *Wnt4*, *Wnt5a*, or *Wnt5b* increases the fractional allocation of MMC neurons, whereas their proportion is reduced in mice that have five mutated *Wnt4*, *Wnt5a*, or *Wnt5b* alleles. (B) Model for the relative contributions of Wnt4/5 and FGF/Hox signaling in the diversification of motor neuron columnar identities. Shh-induced motor neuron progenitors (gray) are exposed to competing signals. Graded Wnt4/5 signals along the DV axis (orange arrow) promote the maintenance of Lhx3 and acquisition of an MMC fate (red). FGF signaling along the AP axis induces differential Hox expression (blue line) so specifying LMC (green) and PGC (brown) fates at limb and thoracic levels, respectively. Some motor neurons at thoracic levels evade Wnt4/5 and FGF-Hox activity and progress to an HMC fate (purple). For details, see text and Dasen et al. (2008).

Over the period that motor neurons acquire their columnar identities, cells in the ventral spinal cord express three noncanonical Wnt genes, *Wnt4*, *Wnt5a*, and *Wnt5b.* The composite profile of these genes appears to establish a ventral-to-dorsal gradient of *Wnt4/5* transcript expression in the ventral spinal cord. Overexpression of *Wnt4/5* increases the generation of MMC neurons, at the expense of segmental columnar classes, whereas reducing *Wnt4/5* expression depletes the spinal cord of MMC neurons and promotes the generation of segmental classes (Figure 7A). These findings indicate that Wnt4/5 signaling is a determinant of MMC identity.

Both Shh and Wnt4/5 signals are graded in character, but there are notable differences in the origin and operation of these gradients. Shh is expressed by floor plate cells, whereas the noncanonical Wnts are expressed over a broader ventral domain, suggesting that the strategy used to establish a Wnt4/5 signaling gradient differs from that underlying the Shh gradient. Shh's pervasive influence on ventral neuronal specification and patterning relies on the long-range spread of Shh protein within the ventral neural epithelium (Jessell, 2000; Chamberlain et al., 2008; Dessaud et al., 2008). In contrast, the range of activity of many secreted Wnts is more limited (Mikels and Nusse, 2006; Hausmann et al., 2007), supporting the idea that the spatial profile of Wnt4/5 activity is established primarily through the graded ventral expression of *Wnt* transcripts. A second difference in the logic of Shh and Wnt4/5 signaling may be the concentration dependence of their activities. Shh functions as a gradient morphogen—specifying distinct ventral cell fates at different concentration thresholds (Jessell, 2000; Dessaud et al., 2008)—whereas the specification of MMC fate could simply require exposure to a critical threshold level of Wnt4/5 signaling. In this view, the graded expression of *Wnt4/5* transcripts may merely serve as an effective strategy for ensuring that an appropriate fraction of cells within the pMN domain are exposed to a threshold level of Wnt4/5 activity.

The slope and spread of the Wnt4/5 activity gradient within the ventral spinal cord appears to be steeper and shorter than that of the Shh gradient. We infer this from the observation that all motor neurons generated in ectopic ventral positions acquire MMC character whereas few if any of the ectopic motor neurons generated dorsal to the normal pMN domain do so. The dorsal limit of Wnt4/5 signaling activity may be constrained by the high level of *Sfrp* expression evident within the p0, p1, and p2 progenitor domains (Kawano and Kypta, 2003; Lei et al., 2006). In this view, the secretion of Sfrp proteins may block the actions of secreted Wnt4/5 proteins that manage to reach these more dorsal domains of the ventral neural tube. The inverted dorsoventral profiles of *Wnt4/5* and *Sfrp* expression are therefore likely to contribute to the restricted range of Wnt4/5 signaling evident within the ventral spinal cord.

How does the Wnt4/5 activity gradient determine the position of generation of motor neuron columnar subtypes within the pMN domain? Were Wnt4/5 protein activity to extend throughout the pMN domain, the probability of generation of neurons of the MMC and segmental motor columns would presumably change smoothly as a function of the dorsoventral position of progenitor cells within the domain. Alternatively, the limit of Wnt4/5 signaling activity could be located within the pMN domain, such that only the most ventrally positioned pMN domain progenitors would have the opportunity to generate MMC neurons. Independent of the linear or step landscape of Wnt4/5 signaling, our findings imply that the diversification of neurons within a single ventral progenitor domain depends on a dorsoventral difference in the intensity or quality of inductive signals (Figure 7A). This position-dependent plan for motor neuron diversification differs conceptually from the mosaic, position-independent mode of Notch signaling that directs to the diversification of certain ventral interneuron subtypes (Peng et al., 2007). Plausibly, the combination of both strategies within a single ventral progenitor domain could further enhance the diversity of neuronal subtypes.

### Wnt4/5 Signaling and the Origins of Spinal Motor Neuron Diversity

Shh and Wnt4/5 signals activate different components of the transcriptional network that controls spinal motor neuron differentiation. Shh signaling is essential for the specification of generic motor neuron character, revealed by the expression of the Nkx6.1/.2, Isl1/2, and Mnr2/Hb9 HD proteins (Chiang et al., 1996; Briscoe et al., 2000; Dessaud et al., 2008). In contrast, Wnt4/5 signaling operates only in the context of a core transcriptional profile established by Shh activity and directs the progression of generic motor neurons to an MMC fate by promoting Lhx3/4 expression. Neural progenitors destined to give rise to other motor neuron columnar subtypes also transiently express Lhx3 (Sharma et al., 1998), indicating that Wnt4/5 signaling specifies MMC fate by programming progenitor cells and/or nascent motor neurons to maintain Lhx3/4 expression after their exit from the cell cycle (Figure 7B).

Why do residual MMC neurons persist under conditions in which five of the six noncanonical *Wnt* alleles have been removed? The activity of the one extant *Wnt4/5* allele could be sufficient to generate a significant number of MMC neurons. Alternatively, additional noncanonical Wnts could still be at work—the early expression of *Wnt11* by axial mesodermal cells (Kispert et al., 1996) could transfer active protein to overlying ventral neural tissue. It is also conceivable that persistent expression of Lhx3 in motor neurons is programmed spontaneously at a low incidence, with Wnt4/5 signaling serving to increase the probability of maintained Lhx3 expression. Defining the transduction pathway through which Wnt4/5 signaling maintains expression of Lhx3 in postmitotic motor neurons may help to distinguish these possibilities. Our findings argue that Wnt4/5 signals are not mediated by canonical β-catenin or planar cell polarity pathways, but leave unresolved the relevant Wnt receptors and intracellular signals.

The discovery that Wnt4/5 signaling specifies MMC character supplies a missing link in the molecular logic of motor neuron columnar diversification and provides a more coherent view of this developmental program. Our findings, together with studies on the specification of LMC and PGC identities (Dasen et al., 2003, 2005, 2008; Rousso et al., 2008), indicate that the program of motor neuron differentiation initiated by Shh signaling generates a set of Hb9^on^, Lhx3^off^ neurons that represent a “ground-state” character of spinal motor neurons. At the time of their generation, this ground-state motor neuron cohort is exposed to two further, opponent, inductive influences: a dorsoventral Wnt4/5 signaling pathway that maintains Lhx3 expression and directs MMC character and a rostrocaudal FGF pathway that patterns Hox expression and so directs LMC and PGC character (Figure 7B). Our data suggest that selection of the Wnt4/5-Lhx3 program precludes neurons from pursuing the FGF-Hox pathway and vice versa. Motor neurons that fail to pursue either of these two options appear to progress to an HMC columnar fate (Dasen et al., 2008; Rousso et al., 2008).

The opponent activities of the Wnt4/5-Lhx3 and FGF-Hox signaling pathways can account for the differing efficiencies of motor neuron columnar interconversion observed under conditions of altered Wnt4/5 signaling. Increasing Wnt4/5 activity results in the conversion of many prospective HMC neurons to an MMC fate. In contrast, prospective LMC and PGC neurons appear to switch fates at much lower efficiency—a consequence of the activation of Hox proteins in these cells (Dasen et al., 2005, 2008). Consistent with this view, the loss of MMC neurons observed at thoracic levels after reductions in Wnt4/5 signaling is accompanied by a preferential increase in HMC, rather than PGC, neurons. This competitive signaling scheme also provides a potential explanation for the finding that enhanced Wnt4/5 signaling does an incomplete job in converting motor neurons to an MMC fate—engagement of the FGF-Hox pathway will have begun to recruit some cells at the time of exposure to Wnt4/5 signals. However, this scheme does not explain why, in *Nkx2.2* mutants, all of the extra motor neurons generated within the former p3 domain acquire MMC character. One possible reason is that the peak of *Wnt4/5* expression in and around the ventral midline results in the activation of Wnt4/5 signaling in the p3 domain at much higher levels than in the pMN domain and thus more effectively recruits cells away from the FGF-Hox option. The high level of Shh signaling activity within the p3 domain could also bias cells in favor of the Wnt4/5-Lhx3 pathway.

Finally, our findings raise the possibility that regulation of Wnt4/5 signaling strength—the operation of a Wnt4/5 rheostat—constitutes a crucial step in transforming the spinal motor system from an MMC-centric plan that typifies early aquatic vertebrates (and larval teleost and amphibian forms) (Fetcho, 1987, 1992) to the more diversified plan of columnar organization and connectivity that characterizes birds and mammals. Conditions of high-level Wnt4/5 signaling may prevail in the ventral neural tube of early aquatic vertebrates, ensuring that most or all Shh-specified motor neurons progress to an Lhx3^on^ MMC-like character. If so, a decrease in the strength of Wnt4/5 signaling may constitute a critical, enabling step in the formation of a population of Shh-specified motor neurons that fail to acquire Lhx3 expression. This Lhx3^off^ motor neuron ground state has recently been shown to serve as the cellular substrate for the columnar programming activities of Hox proteins and FoxP cofactors which direct the formation of LMC and PGC neurons (Dasen et al., 2005, 2008; Rousso et al., 2008).

## Experimental Procedures

### cDNA Probes

Mouse *Wnt1-7b* cDNAs were provided by J. Kitajewski (Columbia University); *Wnt9a-16* cDNAs were amplified by RT-PCR. Chick *Wnt4* was provided by C. Tabin (Harvard University), and chick *Wnt5a* and *Wnt5b* were amplified from total mRNA. Mouse *Wnt1*, -*4*, *-5a*, *-5b*, and -*7b* cDNAs were amplified by PCR and cloned into the pCIG expression vector (Megason and McMahon, 2002). *Fz2*-7 cDNAs were provided by J. Nathans (John Hopkins University); *Fz9* cDNA was provided by U. Francke (Stanford University); *Fz1*, *Fz8*, *Sfrp1*, and *Sfrp2* cDNAs were amplified by RT-PCR. Partial cDNAs for chick *Fz2*, *-3*, *-7*, and -*9* were amplified from HH stage 24 embryos. *Smo^W535L^* cDNA (Hynes et al., 2000) was obtained from L. Zeltser (Columbia University) and cloned into the pCIG expression vector.

### In Situ Hybridization, Immunohistochemistry, and In Ovo Electroporation

In situ hybridization was performed on sections of e9.5 to e10.5 mouse or HH st.18 to 21 chick embryos (Dasen et al., 2003, 2008). Antisense mRNA probes were generated with the DIG RNA Labeling Kit (Roche Applied Science). The graphs for *Wnt* transcript expression were generated with Sigma Plot after measuring the levels of transcript from the ventral midline to the intermediate spinal cord (black arrow) using ImageJ (NIH). The abscissa in these plots represents the average value for each transcript from eight different thoracic sections. The ordinate indicates distance from the ventral midline (microns).

In ovo electroporation into chick neural tube was performed as described (Briscoe et al., 2000). Immunohistochemistry was performed on 15 μm cryostat sections as described (Tsuchida et al., 1994; Dasen et al., 2005). Retrograde labeling of MMC neurons after tracer HRP injection into axial muscles was performed as described (Dasen et al., 2005).

### Mouse Strains

*Nkx2.2*^−/−^, *Wnt4*^−/−^; *Wnt5a*^−/−^, and *Vangl2/Ltap*^−/−^ mice were genotyped as described (Stark et al., 1994; Briscoe et al., 1999; Yamaguchi et al., 1999; Kibar et al., 2001). *Wnt5b*^−/−^ mice were generated by insertion of the PGK-Neo cassette in exon4 using the endogenous PstI and SacI sites followed by gene targeting in ES cells. The wild-type allele of *Wnt5b^+/−^* animals was genotyped with the following primers: Wnt5bwtF (5′GGG ACT CGA ACT CAG ATT GTC AGG3′) and Wnt5bwtR (5′ATG AGC TCG CAG CCG TCC AT3′), located on intron 3 and exon 4, respectively; this generates a 500 bp fragment. The mutant allele was screened with primers: Wnt5bwtF (5′GGG ACT CGA ACT CAG ATT GTC AGG3′) and Wnt5bmutR (5′GCA GGC ATG CTG GGG ATG CGG3′) that amplify a 250 bp band. Animals were housed in the Columbia University Animal Facility and handled according to institutional guidelines.

### Statistical Analysis

Student's t test was used to determine the significance of values of the number of motor neurons and motor columns in experiments where two genotypes or conditions were compared. Mixed effect analysis of variance (ANOVA) was used to test whether variances in the number of total motor neurons or motor columns were different between the wild-type genotype, and the allelic combinations that contained mutated *Wnt4*, *Wnt5a* or *Wnt5b* alleles (a total of 17 genotypes).
