# Supplementary material for: Motor Neurons with Axial Muscle Projections Specified by Wnt4/5 Signaling
Source: Neuron. 2009 Mar 12;61(5):708–20. doi: 10.1016/j.neuron.2008.12.026 (PMC2741579; doi:10.1016/j.neuron.2008.12.026)
Supplement: Document S1. Supplemental Data [file mmc1.pdf]

Neuron, Volume 61

Supplemental Data

Motor Neurons with Axial Muscle Projections Specified by Wnt4/5 Signaling

Dritan Agalliu, Shinji Takada, Ilir Agalliu, Andrew P. McMahon and Thomas M. Jessell

Supplementary Figures and legends

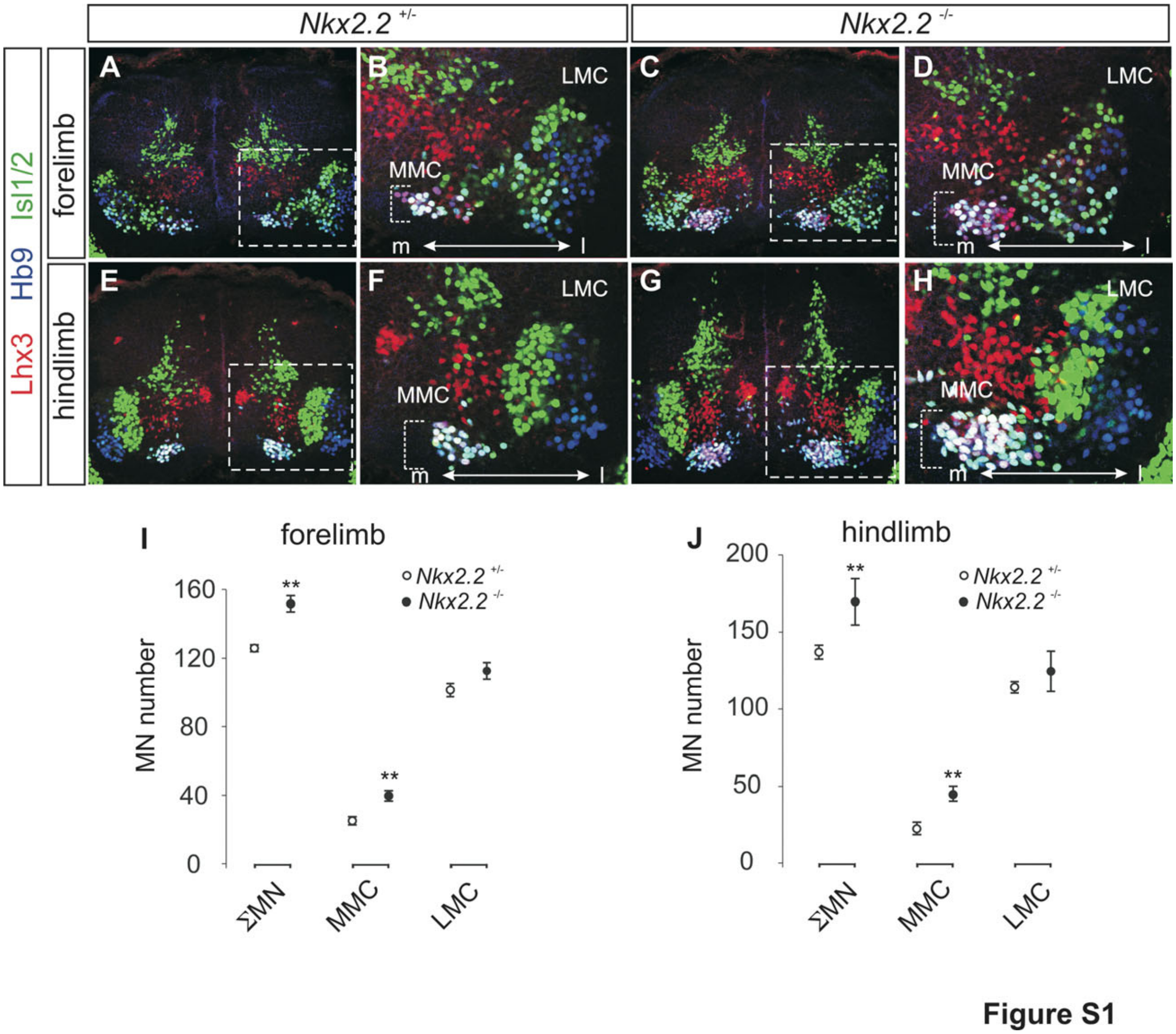

Figure S1. Motor neurons generated from the p3 domain acquire MMC identity at limb levels of the spinal cord.

(A-H). Immunohistochemical labeling for Lhx3 (red), Isl1/2 (green) and Hb9 (blue) in brachial (A-D) and lumbar (E-H) spinal cords from *Nkx2.2* $^{+/-}$ (A, B, E, F) and *Nkx2.2* $^{-/-}$ (C, D, G, H) mice at e13.5. MMC neurons are white (labeled with all three markers); Isl1/2^{+} cells (green) within the motor column are LMCm neurons, whereas Hb9^{+} cells (blue) are LMCl neurons. Note

the increase in the number of MMC neurons (white) in *Nkx2.2* $^{-/-}$ mice. This effect is quantified in the dot plots for forelimb (I) and hindlimb (J) segments, where each circle with bars represents mean ± s.e.m. (n=5 embryos per genotype, Student's t-test, **p<0.01, open circles represent *Nkx2.2* $^{+/-}$ and closed circles are *Nkx2.2* $^{-/-}$).

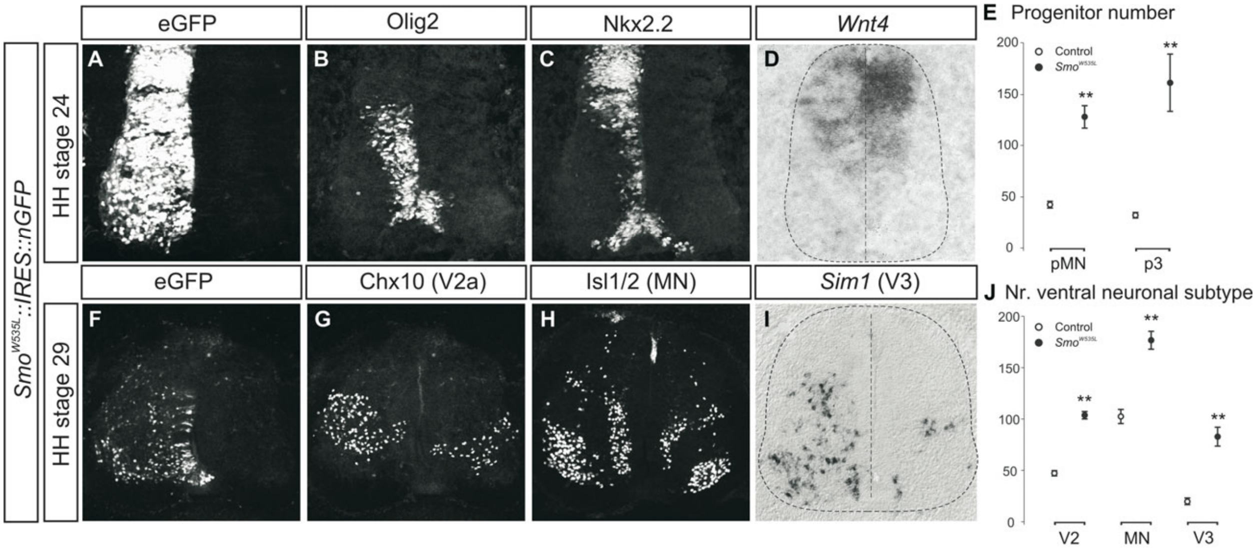

Figure S2

Figure S2. Overexpression of an activated Smoothened receptor expands the pMN domain dorsally and increases all ventral neuronal subtypes.

(A-D) Immunohistochemistry for eGFP (A), Olig2 (B), Nkx2.2 (C), and *in situ* hybridization for *Wnt4* mRNA (D) in *Smo*^{W535L}-transfected chick spinal cord (HH stage 24). Note that misexpression of *Smo*^{W535L}::IRES::nGFP expands dorsally the domain of Olig2 (pMN) and Nkx2.2 (p3) and represses dorsal expression of *Wnt4*. (F-I) Immunohistochemistry for eGFP (F), Chx10 (V2a marker; G), Isl1/2 (MN marker; I) in *Smo*^{W535L}-transfected spinal cords (HH stage 29). (H) *In situ* hybridization with *Sim1* mRNA of *Smo*^{W535L}-transfected spinal cords (HH stage 29) labels V3 interneurons. Note the increase in V2a, MNs and V3 neurons in the transfected side compared to the control. (E, J) Dot plots of the number of progenitors (E) and neuronal subtypes (J) in *Smo*^{W535L}-transfected embryos (mean ± s.e.m; n=8 embryos, Student's t-test, **$p<0.01$, open circle represents control and closed circles *Smo*^{W535L}-transfected sides).

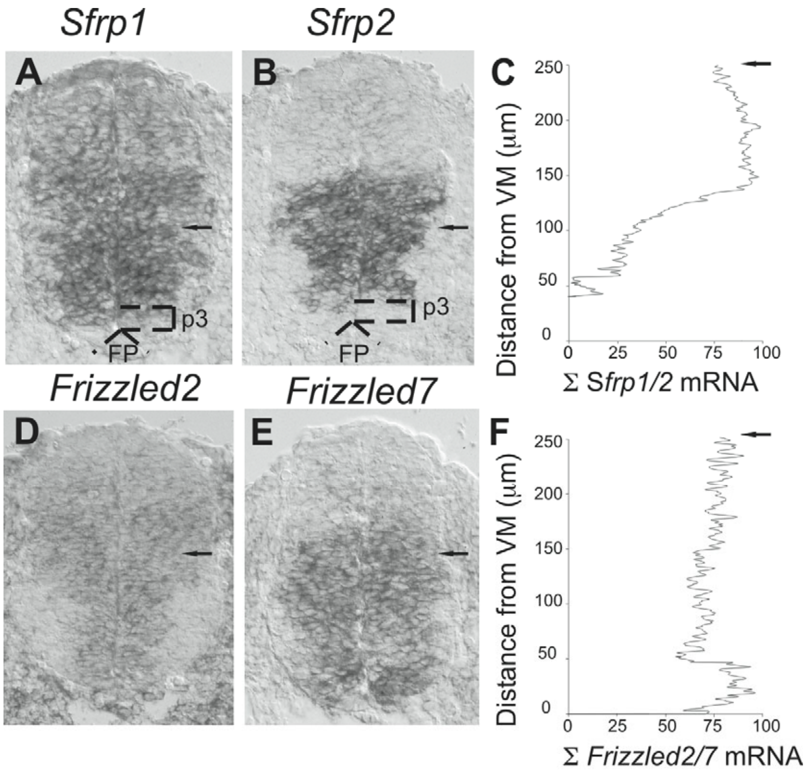

Figure S3

Figure S3. Expression of *Sfrp* and *Frizzled* genes in the ventral spinal cord.

(A, B) *In situ* hybridization with *Sfrp1* (A), *Sfrp2* (B) mRNAs at e9.5 mouse spinal cord. *Sfrp1/2* transcripts are expressed in spinal neural progenitors at the time of motor neuron generation. *Sfrp2* mRNA is excluded from the p3 domain. (D, E) *In situ* hybridization with *Fz2* (D), *Fz7* (E) mRNAs at e9.5 mouse spinal cord. *Fz2* and *Fz7* transcripts are expressed in spinal neural progenitors at the time of motor neuron generation. (C, F) Line plots for the relative amounts of *Sfrp1/2* (C) and *Fz2/7* (H, I) transcripts in the ventral spinal cord. The y-axis represents the relative amount of transcripts, whereas the x-axis represents the distance (μm) from the ventral midline to the intermediate spinal cord (black arrow).

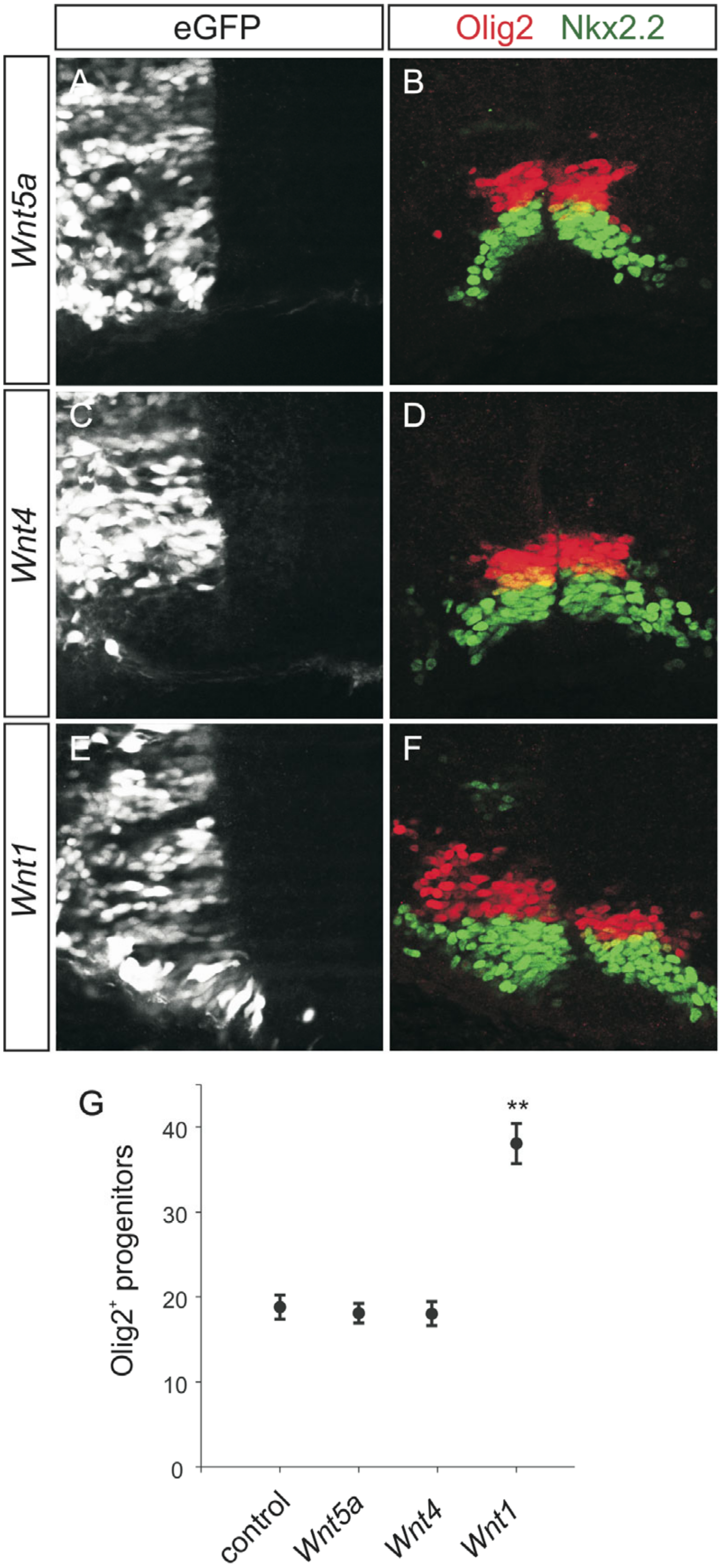
eGFP
Olig2 Nkx2.2
Wnt5a
Wnt4
Wnt1
A
B
C
D
E
F
G
Olig2+ progenitors
40
30
20
10
0
**
control
Wnt5a
Wnt4
Wnt1

Figure S4

Figure S4. Misexpression of Wnt4 or Wnt5a does not affect pMN progenitor proliferation. (A-F) Transfection of spinal cords with *Wnt5a* (A, B), *Wnt4* (C, D) or *Wnt1* (E, F). Immunohistochemistry for eGFP labels the transfected side (A, C, E), Olig2 (red) and Nkx2.2 (green) label pMN and p3 progenitors, respectively (B, D, F). Misexpression of *Wnt5a* or *Wnt4* does not affect pMN or p3 number (B, D). However, transfection of *Wnt1* increases the number of pMN and p3 progenitors (F). (G) Dot plot for pMN progenitor number in control, *Wnt5a*, *Wnt4* or *Wnt1*-transfected hemisegments of the spinal cord. Note the increase in pMN progenitors with *Wnt1* transfection. Each circle with bars represents mean ± s.e.m. (n=6 embryos per genotype, ANOVA test, **$p<0.01$).

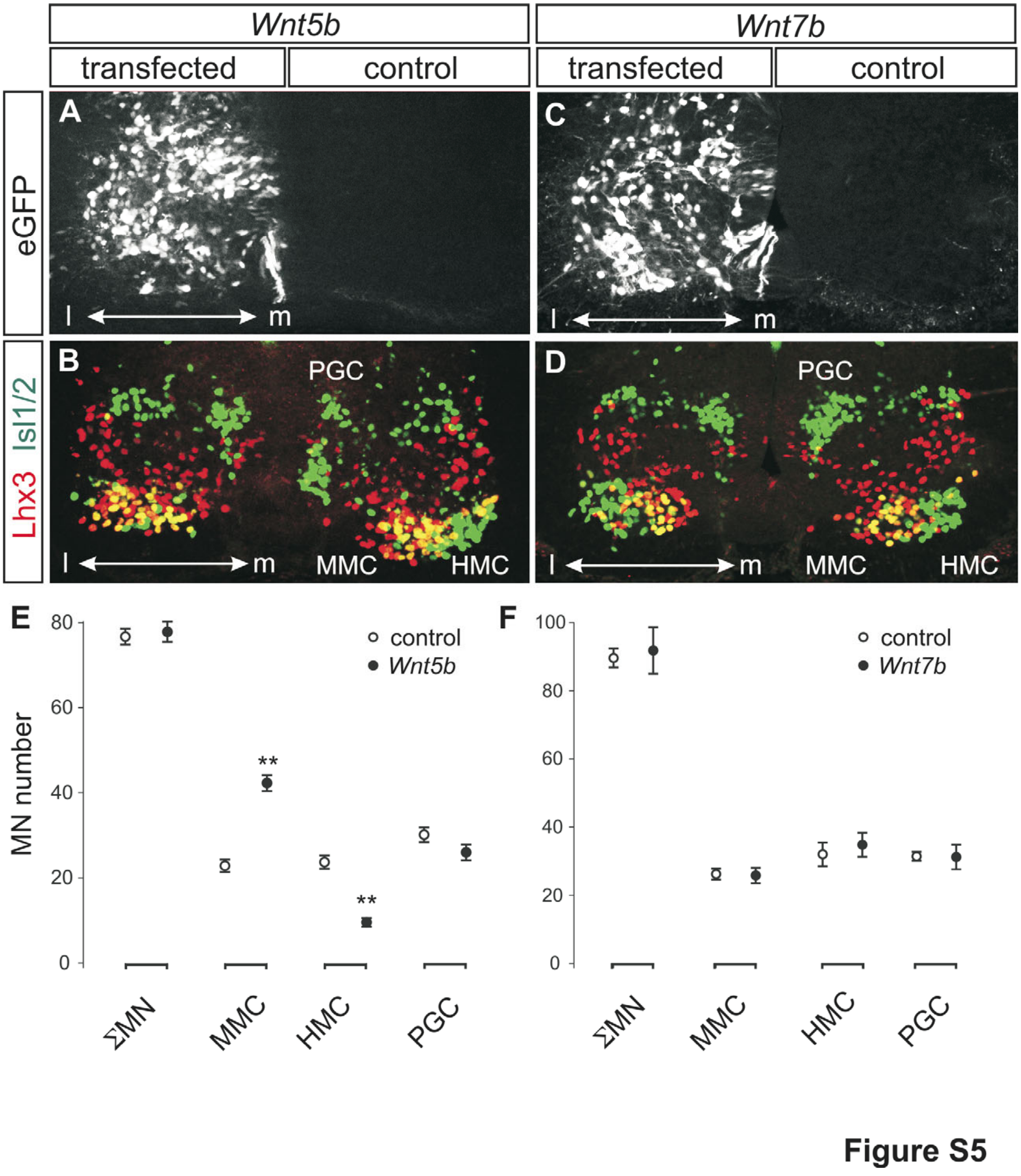

Figure S5. Misexpression of *Wnt5b*, but not *Wnt7b*, promotes MMC fate *in vivo*.

(A-D) Immunohistochemistry for eGFP (A, C) labels the transfected side. Lhx3 (red) and Isl1/2 (green) label MMC neurons (B, D; yellow cells), whereas Isl1/2 marks HMC and PGC columnar

subtypes in embryos transfected with *Wnt5b* (A, B) or *Wnt7b* (C, D). (E, F) Dot plots of the number of motor neurons in *Wnt5b*- (E) or *Wnt7b*-transfected (F) spinal cords. There is an increase in MMC neurons associated with a reduction in HMC neurons in *Wnt5b*-transfected embryos, but no change in the number of motor neurons. *Wnt7b* transfection does not affect motor neurons or columnar subtype allocation. Each circle with bars represents mean ± s.e.m. (Student's t-test; **$p<0.01$; n=5 embryos / transfection).

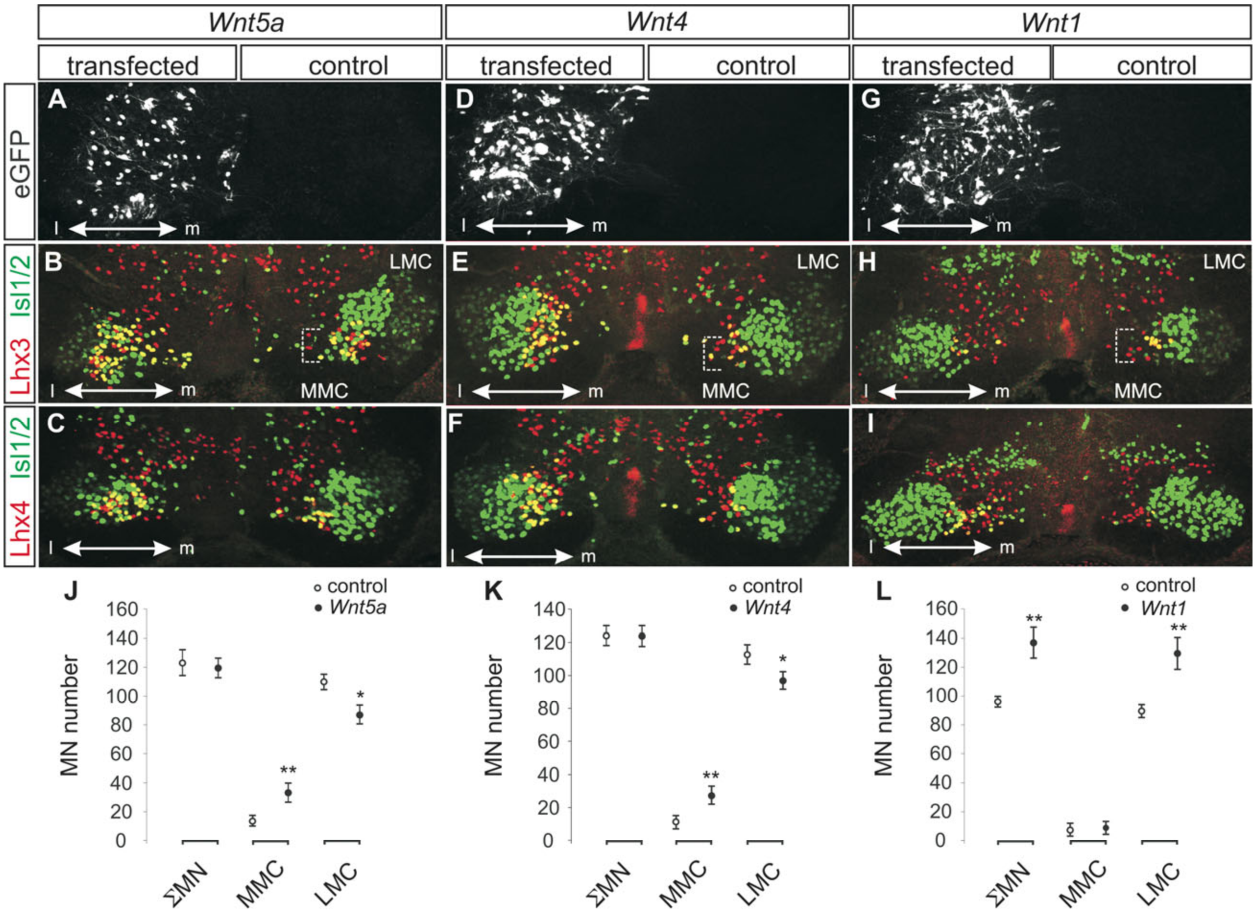

Figure S6

Figure S6. Misexpression of *Wnt4* or *Wnt5a*, but not *Wnt1*, promotes MMC identity at hind-limb level of the spinal cord.

(A-I) Transfection of lumbar spinal cords with *Wnt5a* (A-F), *Wnt4* (G-L) or *Wnt1* (M-R) is marked with eGFP (A, D, G); Lhx3 (red) and Isl1/2 (green) label MMC neurons (B, E, H, yellow cells), whereas Isl1/2 marks LMC columnar subtypes. Misexpression of *Wnt5a* does not affect motor neuron number, but increases the MMC neurons and decreases LMC neurons (B, C, J). *Wnt4* transfection produces similar effects to *Wnt5a* (E, F, K). These ectopic MMC neurons also express Lhx4 (C, F). Transfection of *Wnt1* increases the motor neuron number but not that of MMC neurons (H, I, L). (J-L) Dot plots of the number of motor neurons per hemisection in

Wnt5a (J), *Wnt4* (K) and *Wnt1* (L) transfections. Circles with bars represent mean ± s.e.m (Student's t-test, **$p<0.01$; *$p<0.05$; n=20 embryos for *Wnt5a*, n=16 embryos for *Wnt4*, and n=10 embryos for *Wnt1* transfections; the closed circles represent transfected motor neurons, whereas the open circles are controls).

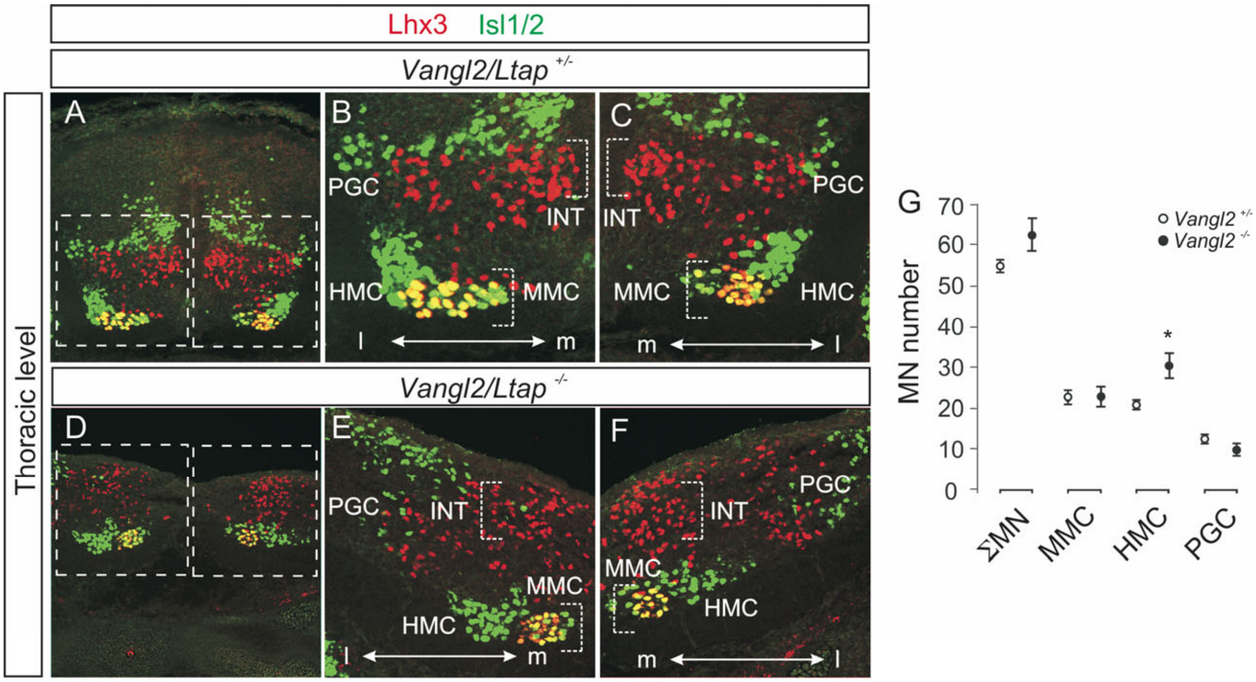

Figure S7

Figure S7. Persistence of MMC identity in mice mutant for the Wnt planar polarity pathway.

(A-F) Lhx3 (red) and Isl1/2 (green) expression in thoracic spinal cord from *Vangl2 (Ltap)*$^{+/-}$ (A-C) and *Vangl2 (Ltap)*$^{-/-}$ (loop tail) (D-F) mice at e13.5. Isl1/2^{on} neurons in the ventral spinal cord are motor neurons (MNs), Lhx3^{on} , Isl1/2^{on} cells are MMC neurons, and Lhx3^{on}, Isl1/2^{off} neurons are V2a interneurons. Note that the neural plate is open in loop tail (*Vangl2/Ltap*) mutant mice. There is no significant change in MMC neuron number, and only a modest change in the number of HMC neurons. (G) Plot of motor neuron numbers per ventral 15 µm quadrant in *Vangl2*$^{+/-}$ (open circles) and *Vangl2*$^{-/-}$ embryos (closed circles); mean ± s.e.m. (n=3 embryos per genotype, Student's t-test, *p<0.05).

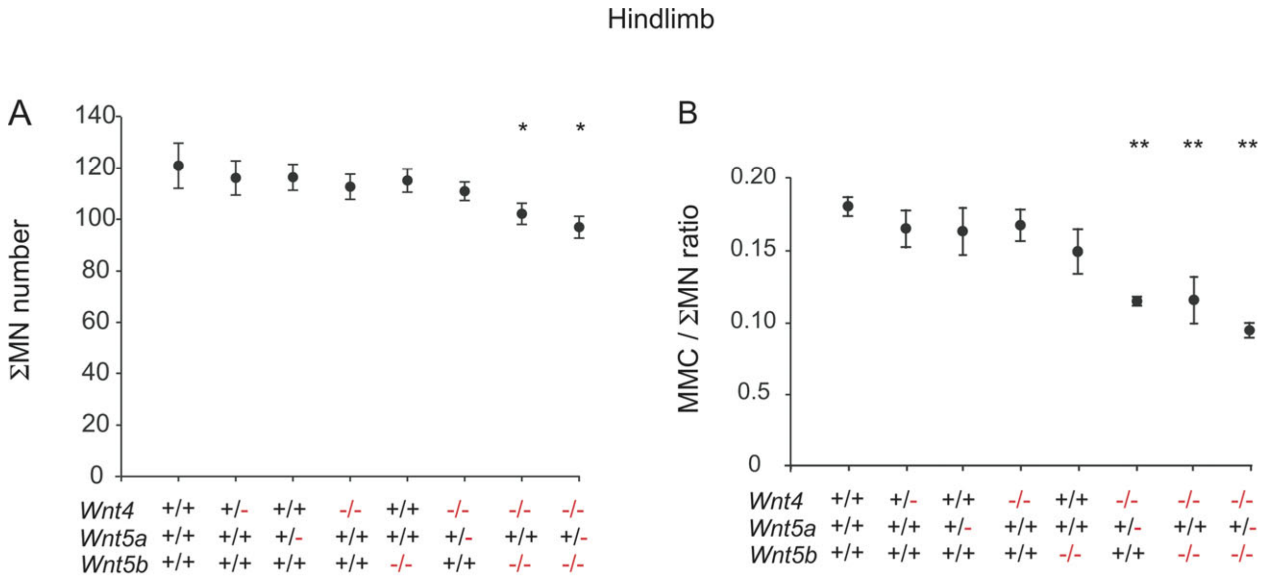

Figure S8

Figure S8. Reduction in MMC ratio at hind-limb levels in *Wnt4/5* mutant mice.

(A-B) Plots of the total number of motor neurons (A) and fraction of MMC over total motor neuron number (B), at lumbar levels, for various allelic combinations of mutant *Wnt4, Wnt5a* and *Wnt5b* genes. The values from embryos with two copies of the mutated *Wnt5a* allele have been omitted because of a dramatic reduction in motor neuron number due to limb defects. There is a ~50% reduction in the ratio of MMC neurons in mice with 4 or 5 mutated *Wnt4*, *Wnt5a* or *Wnt5b* alleles compared to wild type controls. The total number of motor neurons is slightly decreased in mice with 4 or 5 mutated *Wnt4*, *Wnt5a* or *Wnt5b* alleles compared to wild type controls. Circles with error bars represent mean ± s.e.m (ANOVA test, $**p<0.01$, $*p<0.05$, n=3-6 embryos for different genotypes).
